# East Tennessee Dental Association

**Published:** 1871-12

**Authors:** N. I. Mayes


### EAST TENNESSEE DENTAL ASSOCIATION.

The fifth annual meeting of the East Tennessee Dental Association, convened at Knoxville, Tennessee, on Wednesday, September 20th, the President, Dr. Cozier, in the chair.

Several new members were added, and a growing interest was manifested among the members and profession generally.

Dr. Wm. T. Fowler, read an essay on "Mastication and Digestion," which was freely and profitably discussed, as was also one by Dr. Myers, on "Dental Education," and by Dr. Cozier, on "Caries."

Other questions were also discussed, viz.: "Proper Methods of Cleansing Teeth," Treating Exposed Pulp," Flling, Wedging, etc , also Mechanical Dentistry. A clinic was held daily, in which several of the members operated.

Drs. J. Fouche, A. J. Dunn and A. P. White, were appointed a committee to present to the next legislature, a bill prepared by Dr. Cozier, to regulate the practice of dentistry in Tennessee.

The following resolution, presented by Dr. Fouche, was received by the convention:

*Resolved*, That it is the desire of this convention, that two dental chairs be established in connection with the Nashville Medical College, one on operative and one on mechanical dentistry.

*Resolved*, That in our opinion, Dr. Morgan, of Nashville, is the most suitable person to fill the chair of operative dentistry—that we will use our best efforts to procure a charter from the legislature for this purpose.

Drs. Fowler, Mayes and Myers, were appointed a Committee on Membership. The election of officers for the ensuing year then took place, resulting as follows:

*President*—Dr. W. H. Cooke, of Cleveland.

*Vice President*—Dr. A. J. Dunn, of Bristol.

*Corresponding Secretary*—Dr. N. I. Mayes, of Sweetwater.

*Recording Secretary*—Dr. A. P. White, of Knoxville.

A special meeting was appointed to be held at Bristol, on the third Wednesday of June next. The convention then adjourned.

During the session, the highest degree of interest was manifested, and an advancement both in theory and practice was evident. A growing interest in the conventions was also evident, and all left feeling amply rewarded for their attendance, and a seeming determination to use their utmost toward raising the profession in East Tennessee to a high standard.

N. I. MAYES, *Secretary.*

